# Role of Blood Stasis Syndrome of Kampo Medicine in the Early Pathogenic Stage of Atherosclerosis: A Retrospective Cross-Sectional Study

**DOI:** 10.1155/2021/5557392

**Published:** 2021-05-26

**Authors:** Akira Morita, Takao Namiki, Toshiya Nakaguchi, Kazunari Murai, Yuki Watanabe, Michimi Nakamura, Yohei Kawasaki, Yuki Shiko, Yutaka Tamura, Akiko Suganami, Aya Murakami, Akio Yagi, Hideki Okamoto, Yoshiro Hirasaki

**Affiliations:** ^1^Department of Japanese-Oriental (Kampo) Medicine, Graduate School of Medicine, Chiba University, 1-8-1 Inohana, Chuo-ku, Chiba 260-8670, Japan; ^2^Center for Frontier Medical Engineering, Chiba University, 1-33, Yayoi-cho, Inage-ku, Chiba 263-8522, Japan; ^3^Graduate School of Engineering, Chiba University, 1-33, Yayoi-cho, Inage-ku, Chiba 263-8522, Japan; ^4^Biostatistics Section, Clinical Research Center, Chiba University Hospital, 1-8-1 Inohana, Chuo-ku, Chiba 260-8677, Japan; ^5^Department of Bioinformatics, Graduate School of Medicine, Chiba University, 1-8-1 Inohana, Chuo-ku, Chiba 260-8670, Japan; ^6^Center for Pharmaceutical Education, Faculty of Pharmacy, Yokohama University of Pharmacy, 601 Matano-cho, Totsuka-ku, Yokohama 245-0066, Japan

## Abstract

In Kampo medicine, blood stasis (BS) syndrome is strongly associated with microangiopathy and can lead to atherosclerosis. Vascular endothelial dysfunction (VED), evaluated through flow-mediated dilation (FMD), plays an important role in the early stages of atherosclerosis. However, the association of BS syndrome with VED, as determined using FMD, has not been reported. This study investigated the association between BS syndrome and VED using FMD. Forty-one patients with normal glucose tolerance or impaired glucose tolerance (IGT) and without macrovascular complications were evaluated using FMD from May 2017 to August 2017. Based on the BS score, the patients were divided into the non-BS (*n* = 19) and BS syndrome (*n* = 22) groups. Physical and background characteristics, physiological function test results, and laboratory data were compared. Univariate analysis revealed that FMD and a history of dyslipidemia/IGT were significantly different between the two groups (*p* < 0.05). Multiple logistic regression analysis showed that BS syndrome was significantly associated with FMD (odds ratio: 6.26; *p*=0.03) after adjusting for the history of dyslipidemia/IGT. The receiver operating characteristic curve showed that the area under the curve for BS syndrome (0.74; *p* < 0.001) and history of IGT (*p* < 0.007) provided good diagnostic accuracy for FMD. The area under the curve for “BS syndrome + IGT” showed very good accuracy (0.80; *p* < 0.0001) and was higher than that for BS syndrome or IGT alone. In conclusion, the results of this study suggest that the BS score in Kampo medicine could be a useful tool for detecting the early pathogenic stages of atherosclerosis.

## 1. Introduction

Various pathological conditions can cause vascular endothelial dysfunction (VED) in the first stage of atherosclerotic changes. These pathological conditions are often related to lifestyle behaviors, such as an unbalanced diet, lack of sleep or exercise, excessive drinking, stress, and smoking. Moreover, they are recognized as life-threatening owing to the associated risks of cardiovascular and cerebrovascular disease [1, 2].

According to Kampo medicine theory, the human body is composed of three major elements: qi, blood, and fluid. Abnormalities in these elements help define *Mibyou*, the so-called “disease-oriented” healthy stage [3] which can cause subjective symptoms in patients; however, this condition is often not detected by Western medicine due to the absence of abnormal results in routine tests such as blood tests or physiological function tests. In particular, abnormalities in the blood element affect blood stasis (BS), an important pathophysiologic concept that was first documented in the *huangdi neijing*—a medical text that served as a foundation for two other documents, the *shanghan lun* and *jin gui yao lue*,* *and as a subject of many research studies. Based on ancient Chinese medical texts, BS is described as a blood circulation disorder that causes various symptoms, including actual BS, reduced blood flow, and cessation of blood flow [4]. BS is termed *yu xue* in Chinese, *eohyul* in Korean, and *oketsu* in Japanese [5].

Efforts toward a scientific approach in the diagnosis of BS have been unsuccessful, partly owing to the heterogeneity in its assessment by practitioners with varying experience. Therefore, Terasawa et al. advocated the use of the BS score as the basis for the diagnostic criteria of BS (Table 1) [6]. The BS score is based on traditional East Asian medicine text, as well as on previous research. Its data have been analyzed statistically; thus, the BS score is considered as a quantitative standard and is commonly used in various clinical studies on BS syndrome [3–12].

In this study, patients with a total score of less than 21 were classified into the non-blood stasis group, while those with a total score greater than or equal to 21 were classified into the blood stasis syndrome group. Mild symptoms were designated by half points.

BS syndrome is assumed to be associated with microangiopathy [7, 8], which in turn can lead to atherosclerosis [13]. In recent animal studies, the velocity of the microcirculation increased with the administration of *keishibukuryogan* (*Gui-zhi-fu-ling-wan* in Chinese), a Kampo formulation that acts as a BS-ameliorating agent [14].

The ankle-brachial index (ABI) is used mainly for the diagnosis of morphological changes caused by vascular blockage. This index was developed in the mid-1990s, using an oscillometric method [15]. The cardioankle vascular index (CAVI) is a measure of the elastic force of the entire artery, from the origin of the aorta to the ankle. It is used to calibrate the elasticity of a blood vessel to blood pressure using the stiffness parameter *β*, which indicates vascular hardness [15].

VED, which can be evaluated using flow-mediated dilation (FMD), is an early functional change of atherosclerosis. It leads to arterial stiffness, which can be assessed using CAVI, and causes arterial stenosis, which can be evaluated using the ABI. Eventually, this process can result in cardiovascular and cerebrovascular diseases [1, 2]. VED plays a crucial role in the early pathogenic stages of atherosclerosis [16]. Conventional risk factors, such as smoking [17], hypertension [18], dyslipidemia (DLP) [19], and diabetes mellitus [20], have been associated with the severity of VED, and VED has been reported to be an independent risk factor for atherosclerosis [1, 21, 22]. VED has also been associated with microangiopathy in patients with diabetes mellitus [23].

FMD was developed to evaluate the brachial endothelial function and is now widely used as a surrogate marker in clinical trials [24, 25]. FMD is used to study VED using a simple, noninvasive medical ultrasound apparatus designed to measure the change in the forearm vascular diameter caused by the production of nitric oxide. An association of BS syndrome with VED, using FMD, has not been previously reported.

In this study, we investigated the relationship between the BS syndrome of Kampo medicine and VED, as determined by FMD, by examining various parameters including physical and background characteristics, physiological function test results, and laboratory test results of patients who underwent screening for atherosclerosis (Tables S1—S4 in the Supplementary Material).

## 2. Materials and Methods

### 2.1. Patients

**Figure 1:**
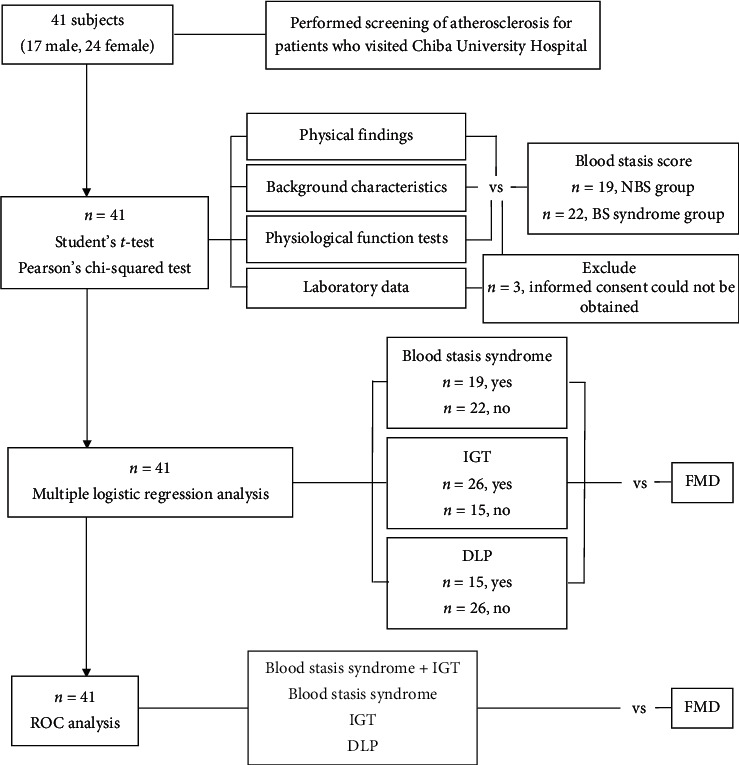
Participant selection. The patients were divided into the NBS group and BS syndrome group. Three patients were excluded from laboratory data analysis because we could not obtain informed consent. The variables found to be associated with the BS score were further tested by multiple logistic regression analysis to investigate the independent factors associated with FMD. To assess the ability of each variable to discriminate FMD, the areas under the ROC curves were calculated. NBS: non-blood stasis; BS: blood stasis; IGT: impaired glucose tolerance; DLP: dyslipidemia; FMD: flow-mediated dilation; ROC; receiver operating characteristic.

This retrospective, cross-sectional study included 41 consecutive patients (17 men and 24 women; average age 70.1 ± 11.9 years; range 33–83 years) with either normal glucose tolerance or impaired glucose tolerance (IGT) and who had no macrovascular complications. Patients were screened for atherosclerosis at the Department of Japanese-Oriental (Kampo) Medicine at Chiba University Hospital between May and August 2017. Twenty-one of the 24 patients in this study were postmenopausal (Figure 1).

### 2.2. Ethical Approval

The study protocol was approved by the Chiba University Hospital Certified Clinical Research Review Board (registration number 1932) on October 6, 2014. Patients provided written informed consent. All processes were conducted in accordance with the tenets of the Declaration of Helsinki (1964).

### 2.3. Classification of the BS Score

Five practitioners of Kampo medicine, with ≥10 years of experience in clinical practice [26], classified the 41 patients into the non-BS (NBS) group and BS syndrome group based on the BS score. This diagnostic procedure was conducted as per standard clinical practice. The BS score contains 17 evaluation items, with total scores ranging from 0 to 90 points for men and from 0 to 101 points for women. Regardless of sex, patients with total scores <21 were classified into the NBS group and those with total scores ≥21 were classified into the BS syndrome group (Table 1).

### 2.4. Physical and Background Characteristics

Physical characteristics obtained from the patients' medical records included age, sex, height, and body weight; the two latter characteristics were used to calculate the body mass index (BMI = body weight (in kg)/height^2^ (in meters)). Data on systolic blood pressure (SBP), diastolic blood pressure (DBP), and background characteristics such as smoking history and past medical history were also collected.

## 3. FMD

Ultrasound studies to evaluate endothelial function were conducted from approximately 8:00 AM to 12:00 PM in a temperature-controlled room (22–26°C), with the patient in a fasting, resting, and supine position state. Meals and all types of beverages, except water, were prohibited from the night before the study. The diameter of the patient's brachial artery was measured according to the Japan Circulation Society (JCS) guidelines for noninvasive vascular function tests (JCS 2013) [15]. A semiautomatic ultrasound device (UNEXEF 38 G; UNEX, Nagoya, Japan) was used to determine the vasodilator responses of the brachial artery.

Avascularization was performed by inflating a blood pressure cuff to SBP + 50 mm Hg for 5 min. An arterial image was obtained at approximately 1 min after deflation. The FMD was quantified as the percentage change in brachial artery diameter within 1 min after avascularization, relative to the baseline vessel diameter. As in previous studies, the cutoff value of FMD was set at 5% [27, 28]. The VED was constantly evaluated by monitoring the FMD.

### 3.1. Physiological Function Tests for Atherosclerosis

Physiological function tests (i.e., ABI and CAVI) were performed 10 min after measuring the FMD, according to the guidelines for noninvasive vascular function testing [15]. We added the right and left measured values and calculated the average value to determine the ABI and CAVI values (VaSera VS-1500N; Fukuda Denshi, Tokyo, Japan).

### 3.2. Laboratory Data

Informed consent could not be obtained from three patients; therefore, blood tests were conducted on 38 out of the 41 patients at the hospital's clinical laboratory department on the same day after the physiological function testing. We used the clinical laboratory data for triglycerides, high-density lipoprotein cholesterol, low-density lipoprotein cholesterol, hemoglobin A1c, fasting plasma glucose (FPG), and immunoreactive insulin (IRI). The latter two were used to calculate the homeostasis model assessment ratio (HOMA-R):

(1) $$\begin{array}{c} \text{HOMA-R }=\text{ IRI }\left[\frac{\mu U}{\text{mL}}\right]\;\times \text{ FPG}\frac{\left[\text{mg}/\text{dL}\right]}{405}, \end{array}$$

and homeostasis model assessment beta-cell function (HOMA-*β*):

(2) $$\begin{array}{c} \text{HOMA-}\beta \;=\text{ FPG }\left[\frac{\text{mg}}{\text{dL}}\right]\times \;\frac{360}{\text{IRI}}\left[\frac{\mu \text{U}}{\text{mL}}\right]-63\text{ ,} \end{array}$$

which are used to evaluate whether the patient had normal glucose tolerance or IGT. High-sensitivity C-reactive protein (hsCRP) levels were measured by standard laboratory techniques at our hospital and at the Special Reference Laboratories (Tokyo, Japan).

### 3.3. Statistical Analysis

The results are expressed as the number and percentage of cases for discrete variables and the mean ± standard deviation for continuous variables. For assessment of patient characteristics, Student's *t*-test was applied when the variables were continuous, and Pearson's chi-squared test was used when the variables were discrete.

The variables found to be associated with the BS score were further tested by multiple logistic regression analysis to investigate the independent factors associated with FMD. The multiple logistic regression analysis was also used to determine the odds ratios (ORs) and the 95% confidence intervals for BS syndrome as a factor associated with FMD compared with NBS.

To assess the ability of each variable to discriminate FMD, the areas under the receiver operating characteristic (ROC) curves (AUCs) were calculated. In addition to the asymptotic 95% confidence interval, *p* values under the null hypothesis (true area = 0.50) were calculated. An AUC of >0.9 was considered excellent; 0.8–0.9, very good; 0.7–0.8, good; 0.6–0.7, average; and <0.6, poor [29, 30]. The significance level was set at <5% in each analysis. All statistical analyses of the recorded data were performed using the Excel statistical software package (Statcel 4; OMS Publishing Inc., Saitama, Japan) and SAS statistical software package, version 9.4 (SAS Institute, Cary, NC, USA).

## 4. Results

### 4.1. Patient Characteristics

Baseline characteristics of the patients in the NBS and BS syndrome groups who completed all the tests are summarized in Table 2. The BS syndrome group had a significantly lower FMD (4.9 ± 1.4 vs. 3.3 ± 1.7, *p* < 0.01) than the NBS group; moreover, the BS syndrome group had a significantly higher prevalence of a history of DLP/IGT (*p* < 0.05/*p* < 0.01) than the NBS group. Other variables were not significantly different between the groups.

### 4.2. Laboratory Data

Laboratory data of 38 patients out of the entire study population are summarized by group in Table 3. No significant differences were found between the groups.

### 4.3. Factors Associated with FMD

A multiple logistic regression analysis showed that BS syndrome was significantly associated with FMD (OR: 6.26; *p*=0.03) after adjusting for a history of DLP and IGT, both of which were not significant in the model (Table 4). The OR indicates that BS syndrome has a greater risk than NBS.

**Figure 2:**
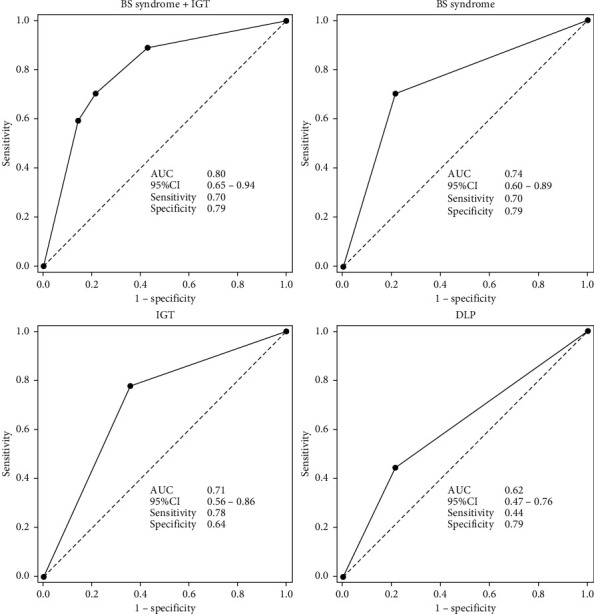
The diagnostic accuracy of blood stasis (BS) syndrome + impaired glucose tolerance (IGT), BS syndrome alone, IGT alone, and dyslipidemia (DLP) for predicting flow-mediated dilation (FMD). The receiver operating characteristic (ROC) curves depict that BS syndrome and IGT have good diagnostic utility for FMD, with BS syndrome + IGT having better diagnostic accuracy; DLP demonstrates poor diagnostic utility. In each graph, the solid diagonal line is the line of no discrimination (true area = 0.50), and the optimal cutoff points are indicated on the curves. AUC: area under the curve; CI: confidence interval.

ROC curves were generated using BS syndrome, DLP, and IGT to determine whether they could distinguish NBS patients from BS syndrome patients. BS syndrome and IGT showed modest utility, with the ROC curves being higher and shifted to the left compared to DLP, which had poor fitting and utility. The AUCs of BS syndrome and IGT individually showed a good fit (0.74 and 0.71, respectively) and were not significantly different from each other. However, the AUC for DLP was 0.62, which failed to reject the null hypothesis (true area = 0.50). We performed multiple logistic regression to identify a better discriminator of FMD. We set the model using FMD as a dependent variable and the combination of BS syndrome and IGT as a single independent variable (“BS syndrome + IGT”). The AUC of the combined “BS syndrome + IGT” parameter (0.80) showed a better fit than that of either BS syndrome or IGT (Table 5 and Figure 2).

## 5. Discussion

In this study, ROC analysis indicated that the AUCs of BS syndrome and IGT showed a fair diagnostic accuracy for FMD. Furthermore, the AUC of the combined “BS syndrome + IGT” parameter showed better diagnostic accuracy than that of BS syndrome or IGT. The AUCs of BS syndrome and IGT were 0.74 and 0.71, respectively, thereby demonstrating a very good diagnostic accuracy for predicting FMD in patients at low risk for cardiovascular disease. Although there were no significant differences between these outcome measures when comparing the AUC, the multiple logistic regression analysis showed that BS syndrome was a significant factor for FMD after adjusting for DLP and IGT, neither of which were significant in the model. Thus, the findings of this study suggest that BS syndrome might be more useful than IGT for discerning the presence of FMD in patients who are at low risk for cardiovascular events.

From the results of the multiple logistic regression analysis, the AUC of the combined parameter “BS syndrome + IGT” demonstrated better accuracy than that of BS syndrome or IGT alone. This suggests that BS syndrome combined with IGT may be a better discriminator of VED, as determined by FMD than by BS syndrome alone.

An association between BS syndrome and atherosclerotic changes based on the ABI [31] and the CAVI [5] has been reported; moreover, studies have shown a relationship between atherosclerotic changes and VED [27]. However, an association between BS syndrome and VED, as determined by FMD, has not been previously reported. In our study, contrary to the FMD results, the ABI and CAVI results were not significantly different between the groups.

The findings of this study should be considered in the light of three caveats. First, it should be considered that a lack of an association between BS syndrome and the physiological function tests (ABI and CAVI) may be explained by selecting patients who had a lower risk of cardiovascular events than those in previous studies [5]. Second, FMD was significantly lower for patients who had IGT with DLP, even though significant changes in ABI and CAVI were not found [32]. Results from previous studies and our study (shown in Table 2) are similar; therefore, a causal relationship of BS syndrome with VED can be considered plausible. Third, although sex-related differences in VED are thought to be related to age and hormonal status [33, 34], our findings indicate that there is no significant difference in FMD between male and female patients.

These results suggest that BS syndrome can represent a lower-grade abnormal functional change, and hence, arterial stiffness and stenosis represent morphological changes. Therefore, the diagnosis of BS syndrome in Kampo medicine can reflect early phases of atherosclerosis in patients with a relatively low risk of cardiovascular events.

In addition to our research, BS has been reported to be associated with atherosclerosis [35] and cardiovascular disease [36]. Both BS and VED are suspected to be associated with microangiopathy and atherosclerosis. Furthermore, VED is considered reversible [1]. Thus, early intervention and prevention are of critical importance. Improved FMD has been observed after the administration of hypotensive [37], hypolipidemic [38], and hypoglycemic agents [39], as well as with the implementation of exercise therapy [40], dietetic therapy [41], and smoking cessation [42]. *Keishibukuryogan* (*Gui-zhi-fu-ling-wan* in Chinese) has been shown to improve VED [43], which is considered to be the early phase of atherosclerosis. Zu San Li (ST36) and Nei Guan (PC6) in their randomized double-blind study showed an improvement in VED, as evaluated with FMD [44], using acupuncture.

Therefore, the findings of this study suggest that the diagnosis of BS syndrome may enable the implementation of lifestyle modifications, contributing to early intervention in the early stages of atherosclerosis. This will be of particular interest to medical staff in remote locations, disaster zones, and developing countries where patients may not be accessed to advanced medical care.

This study had several limitations. First, the ROC curves were difficult to interpret owing to the small sample size (*n* = 41). Second, although precedents exist in previous studies, the definition of cutoff values for FMD was arbitrary. Third, studies comparing arterial condition and blood stasis in this field are still few. Blood stasis in Kampo medicine includes the dysfunction of blood distribution not only in arteries but also in veins and capillaries. This study comprises only one of the components of blood stasis. Thus, validation with larger cohorts, methodological improvement, and strictly defined protocols are necessary for future studies.

## 6. Conclusions

Our study indicates that BS syndrome is a significant factor for VED, as determined by FMD. Furthermore, a superior diagnostic accuracy can be obtained by combining BS score with IGT. The findings of this study suggest that the BS syndrome in Kampo medicine can be a useful tool to detect early pathogenic stages of atherosclerosis.

## Figures and Tables

**Table 1:** Diagnostic criteria for blood stasis syndrome.

Symptom	Score	
	Males	Females
Dark-rimmed eyes	10	10
Areas of dark pigmentation of facial skin	2	2
Rough skin	2	5
Livid lips	2	2
Livid gingiva	10	5
Livid tongue	10	10
Telangiectasis/vascular spiders	5	5
Subcutaneous hemorrhage	2	10
Palmar erythema	2	5
Resistance and tenderness on pressure of the left paraumbilical region	5	5
Resistance and tenderness on pressure of the right paraumbilical region	10	10
Resistance and tenderness on pressure of the umbilical region	5	5
Resistance and/or tenderness on pressure of the ileocecal region	5	2
Resistance and/or tenderness on pressure of the sigmoidal region	5	5
Resistance and/or tenderness on pressure of the subcostal region	5	5
Hemorrhoids	10	5
Dysmenorrhea	—	10
Total score	90	101

**Table 2:** Patient characteristics of the NBS group and BS syndrome group (*n* = 41).

Variables	Units	NBS group (*n* = 19)	BS syndrome group (*n* = 22)	*p* value
Age	Years	64.2 ± 12.9	70.9 ± 10.6	0.06^a^
Sex	Male/female	5/14	12/10	0.07^b^
BMI	kg/m^2^	22.6 ± 3.8	24.4 ± 3.5	0.13^a^
SBP	mmHg	128.4 ± 19.3	134.5 ± 19.3	0.33^a^
DBP	mmHg	74.3 ± 8.8	70.4 ± 13.6	0.30^a^
Smoking	Yes/no	5/14	9/13	0.33^b^
Hypertension	Yes/no	8/11	13/9	0.28^b^
DLP	Yes/no	3/16	12/10	<0.05^b^
Cardiovascular disease	Yes/no	1/18	3/19	0.37^b^
Cerebrovascular disease	Yes/no	3/16	4/18	0.84^b^
IGT	Yes/no	8/11	18/4	<0.01^b^
Diabetic complications				
Neuropathy	Yes/no	1/18	2/20	0.64^b^
Retinopathy	Yes/no	0/19	2/20	0.18^b^
Nephropathy	Yes/no	0/19	2/20	0.18^b^
Antithrombotic drug	Yes/no	8/11	13/9	0.28^b^
ABI		1.10 ± 0.08	1.04 ± 0.11	0.07^a^
CAVI		8.4 ± 1.5	9.2 ± 1.2	0.07^a^
FMD	%	4.9 ± 1.4	3.3 ± 1.7	<0.01^a^

NBS: non-blood stasis; BS: blood stasis; BMI: body mass index; SBP: systolic blood pressure; DBP: diastolic blood pressure; DLP: dyslipidemia; IGT: impaired glucose tolerance; ABI: ankle-brachial index; CAVI: cardioankle vascular index; FMD: flow-mediated dilation. ^a^Comparison between the groups by Student's *t*-test. ^b^Comparison between the groups by Pearson's chi-squared test. Data are expressed as the mean ± standard deviation.

**Table 3:** Blood test data of the NBS group and BS syndrome group (*n* = 38).

Variables	Units	NBS group (*n* = 18)	BS syndrome group (*n* = 20)	*p* value
TG	mg/dL	114.4 ± 63.0	123.4 ± 61.4	0.67^a^
HDL cholesterol	mg/dL	65.6 ± 21.0	56.7 ± 11.3	0.12^a^
LDL cholesterol	mg/dL	122.8 ± 41.1	118.9 ± 31.7	0.75^a^
HbA1c	NGSP, %	6.2 ± 0.9	6.6 ± 0.8	0.21^a^
FPG	mg/dL	113.9 ± 24.6	125.9 ± 31.6	0.22^a^
IRI	*μ*U/mL	4.3 ± 2.4	8.8 ± 10.0	0.08^a^
HOMA-R		1.3 ± 0.8	3.4 ± 5.4	0.11^a^
HOMA-*β*	%	34.5 ± 26.2	42.3 ± 29.7	0.41^a^
hsCRP	mg/dL	0.11 ± 0.18	0.31 ± 0.52	0.13^a^

NBS: non-blood stasis; BS: blood stasis; TG: triglyceride; HDL: high-density lipoprotein; LDL: low-density lipoprotein; HbA1c: hemoglobin A1c; FPG: fasting plasma glucose; IRI: immunoreactive insulin; HOMA-R: homeostasis model assessment ration; HOMA-*β*: homeostasis model assessment of beta-cell function; hsCRP: high-sensitivity C-reactive protein. ^a^Comparison between the groups by Student's *t*-test. Data are expressed as the mean ± standard deviation.

**Table 4:** Multiple logistic regression analysis for FMD as a dependent variable and BS syndrome, IGT, and DLP as independent variables (*n* = 41).

Variables		OR	95% CI	*p* value
BS syndrome	≥21	6.26	1.17–33.52	0.03
	<21	Reference		

IGT	Yes	4.12	0.73–23.06	0.11
	No	Reference		

DLP	Yes	0.75	0.10–5.49	0.78
	No	Reference		

FMD: flow-mediation dilation; BS: blood stasis; IGT: impaired glucose tolerance; DLP: dyslipidemia; OR: odds ratio; CI: confidence interval. FMD < 5%.

**Table 5:** Areas under the curves for BS syndrome + IGT, BS syndrome, IGT, and DLP (*n* = 41).

Variables	Sensitivity	Specificity	AUC	SE	95% CI	*p* value
BS syndrome + IGT	0.70	0.79	0.80	0.07	0.65–0.94	<0.0001
BS syndrome	0.70	0.79	0.74	0.07	0.60–0.89	0.001
IGT	0.78	0.64	0.71	0.08	0.56–0.86	0.007
DLP	0.44	0.79	0.62	0.07	0.47–0.76	0.125

BS: blood stasis; IGT: impaired glucose tolerance; DLP: dyslipidemia; AUC: area under the curve; CI: confidence interval; SE: standard error.

## Data Availability

The data used to support the findings of this study are available within the article and its supplementary materials.

## Supplementary Materials

Supplementary Materials Table S1: physical findings. Table S2: background characteristics. Table S3: background characteristics and results of physiological function tests. Table S4: laboratory test results.
