# Supplementary material for: Role of Blood Stasis Syndrome of Kampo Medicine in the Early Pathogenic Stage of Atherosclerosis: A Retrospective Cross-Sectional Study
Source: Evid Based Complement Alternat Med. 2021 May 26;2021:5557392. doi: 10.1155/2021/5557392 (PMC8175131; doi:10.1155/2021/5557392)
Supplement: Supplementary Materials — Table S1: physical findings. Table S2: background characteristics. Table S3: background characteristics and results of physiological function tests. Table S4: laboratory test results. [file 5557392.f1.docx]

## Supplementary Materials

**Table S1: Physical findings**

| n = 41 |  |  |  |  |  |  |
| --- | --- | --- | --- | --- | --- | --- |
|  |  |  |  |  |  |  |
| Patient No. | BS Score | Age  (years) | Sex | BMI  (kg/m^2^) | SBP  (mmHg) | DBP  (mmHg) |
| 1 | 17.0 | 64 | F | 16.2 | 132 | 81 |
| 2 | 20.0 | 36 | F | 17.8 | 104 | 61 |
| 3 | 42.0 | 84 | F | 20.0 | 141 | 65 |
| 4 | 26.0 | 86 | F | 27.8 | 120 | 40 |
| 5 | 52.0 | 79 | F | 29.3 | 138 | 68 |
| 6 | 25.0 | 69 | M | 21.1 | 161 | 79 |
| 7 | 50.0 | 73 | M | 22.2 | 129 | 73 |
| 8 | 37.0 | 77 | F | 24.0 | 137 | 77 |
| 9 | 7.0 | 82 | F | 20.1 | 116 | 68 |
| 10 | 45.0 | 61 | M | 25.0 | 162 | 81 |
| 11 | 42.0 | 84 | M | 21.4 | 176 | 86 |
| 12 | 39.0 | 79 | F | 26.2 | 122 | 80 |
| 13 | 10.0 | 42 | F | 29.9 | 129 | 80 |
| 14 | 14.5 | 71 | F | 19.0 | 125 | 70 |
| 15 | 39.0 | 78 | F | 29.3 | 142 | 52 |
| 16 | 24.0 | 77 | M | 26.6 | 142 | 70 |
| 17 | 19.0 | 75 | M | 24.2 | 133 | 76 |
| 18 | 16.0 | 77 | F | 27.2 | 111 | 65 |
| 19 | 51.0 | 83 | M | 21.4 | 141 | 81 |
| 20 | 12.0 | 67 | F | 18.3 | 152 | 85 |
| 21 | 12.0 | 70 | M | 20.5 | 125 | 79 |
| 22 | 19.5 | 60 | F | 29.4 | 144 | 81 |
| 23 | 27.0 | 77 | M | 25.3 | 113 | 63 |
| 24 | 19.5 | 70 | F | 21.5 | 113 | 62 |
| 25 | 14.0 | 84 | M | 24.3 | 136 | 89 |
| 26 | 17.0 | 76 | F | 25.5 | 120 | 65 |
| 27 | 39.0 | 51 | M | 25.8 | 116 | 83 |
| 28 | 14.5 | 59 | F | 21.3 | 99 | 63 |
| 29 | 44.0 | 75 | M | 23.9 | 143 | 89 |
| 30 | 27.0 | 79 | F | 26.0 | 131 | 82 |
| 31 | 9.0 | 78 | M | 19.5 | 128 | 72 |
| 32 | 17.0 | 82 | F | 25.4 | 145 | 80 |
| 33 | 27.0 | 67 | F | 29.3 | 137 | 82 |
| 34 | 35.0 | 66 | F | 26.1 | 148 | 80 |
| 35 | 42.0 | 83 | M | 20.2 | 125 | 61 |
| 36 | 12.0 | 58 | F | 21.4 | 126 | 78 |
| 37 | 59.0 | 82 | M | 19.8 | 103 | 47 |
| 38 | 17.0 | 55 | F | 25.4 | 187 | 89 |
| 39 | 44.0 | 80 | M | 29.0 | 145 | 58 |
| 40 | 12.0 | 71 | M | 22.3 | 114 | 68 |
| 41 | 44.0 | 46 | F | 16.5 | 88 | 51 |

BS: blood stasis; M, male; F, female; BMI: body mass index; SBP: systolic blood pressure; DBP: diastolic blood pressure

**Table S2: Background characteristics**

| n = 41 |  |  |  |  |  |  |
| --- | --- | --- | --- | --- | --- | --- |
|  |  |  |  |  |  |  |
| Patient No. | Smoking | Hypertension | DLP | Cardiovascular disease | Cerebrovascular disease | IGT |
| 1 | - | - | - | - | - | - |
| 2 | - | - | - | - | - | - |
| 3 | - | ○ | - | ○ | ○ | ○ |
| 4 | - | - | ○ | - | - | ○ |
| 5 | - | ○ | ○ | - | - | ○ |
| 6 | - | ○ | ○ | - | - | ○ |
| 7 | ○ | ○ | - | - | - | ○ |
| 8 | - | - | ○ | - | - | ○ |
| 9 | - | ○ | - | - | - | ○ |
| 10 | ○ | - | - | - | - | ○ |
| 11 | ○ | ○ | - | - | - | ○ |
| 12 | - | ○ | ○ | - | - | ○ |
| 13 | ○ | - | ○ | - | - | ○ |
| 14 | - | - | - | - | - | ○ |
| 15 | - | ○ | ○ | - | - | ○ |
| 16 | - | - | - | - | - | ○ |
| 17 | ○ | - | - | - | - | ○ |
| 18 | - | - | - | - | - | - |
| 19 | - | ○ | - | - | - | - |
| 20 | - | - | - | - | - | - |
| 21 | - | ○ | - | - | - | - |
| 22 | - | ○ | - | - | - | ○ |
| 23 | ○ | ○ | ○ | - | - | ○ |
| 24 | - | ○ | ○ | ○ | - | ○ |
| 25 | ○ | ○ | - | - | - | - |
| 26 | - | ○ | - | - | - | ○ |
| 27 | - | - | ○ | - | - | ○ |
| 28 | - | - | - | - | ○ | - |
| 29 | ○ | ○ | ○ | - | - | ○ |
| 30 | - | ○ | ○ | ○ | ○ | ○ |
| 31 | ○ | - | - | - | - | ○ |
| 32 | - | ○ | ○ | - | ○ | - |
| 33 | - | ○ | ○ | - | - | ○ |
| 34 | - | - | - | - | - | - |
| 35 | ○ | - | - | - | ○ | - |
| 36 | - | - | - | - | - | - |
| 37 | ○ | - | - | - | - | ○ |
| 38 | - | ○ | - | - | ○ | - |
| 39 | ○ | ○ | ○ | ○ | ○ | ○ |
| 40 | ○ | - | - | - | - | - |
| 41 | ○ | - | - | - | - | - |

DLP: dyslipidemia; IGT: impaired glucose tolerance.

**Table S3: Background characteristics and results of physiological function tests**

| n = 41 |  |  |  |  |  |  |  |
| --- | --- | --- | --- | --- | --- | --- | --- |
|  | Diabetic complications | | |  |  |  |  |
| Patient No. | Neuropathy | Retinopathy | Nephropathy | Antithrombotic drug | ABI | CAVI | FMD  (%) |
| 1 | - | - | - | - | 1.07 | 8.5 | 6.5 |
| 2 | - | - | - | - | 0.98 | 6.1 | 5.5 |
| 3 | - | - | - | ○ | 1.09 | 10.6 | 1.7 |
| 4 | - | - | - | - | 1.11 | 9.0 | 3.8 |
| 5 | - | - | ○ | ○ | 1.15 | 8.7 | 3.4 |
| 6 | ○ | ○ | - | ○ | 0.84 | 8.0 | 0.8 |
| 7 | - | - | - | ○ | 1.16 | 10.3 | 2.6 |
| 8 | - | - | - | - | 1.11 | 8.5 | 4.5 |
| 9 | - | - | - | ○ | 0.99 | 7.7 | 3.1 |
| 10 | - | - | - | - | 1.10 | 7.3 | 4.5 |
| 11 | - | - | - | ○ | 1.11 | 9.5 | 3.5 |
| 12 | - | - | - | ○ | 1.04 | 8.4 | 2.4 |
| 13 | - | - | - | - | 1.00 | 6.1 | 5.3 |
| 14 | - | - | - | - | 1.14 | 10.1 | 3.7 |
| 15 | - | - | - | ○ | 1.09 | 9.2 | 3.3 |
| 16 | - | - | - | - | 1.08 | 11.2 | 2.3 |
| 17 | - | - | - | - | 1.18 | 9.9 | 7.0 |
| 18 | - | - | - | - | 1.16 | 7.8 | 3.9 |
| 19 | - | - | - | ○ | 0.80 | 12.2 | 0.8 |
| 20 | - | - | - | - | 1.00 | 8.2 | 5.8 |
| 21 | - | - | - | ○ | 1.30 | 9.1 | 5.1 |
| 22 | - | - | - | ○ | 1.11 | 8.1 | 2.6 |
| 23 | ○ | - | - | ○ | 0.92 | 8.7 | 6.7 |
| 24 | - | - | - | ○ | 1.11 | 9.8 | 3.1 |
| 25 | - | - | - | ○ | 1.14 | 10.6 | 4.4 |
| 26 | - | - | - | ○ | 1.10 | 9.3 | 3.0 |
| 27 | - | - | - | - | 1.08 | 8.8 | 5.4 |
| 28 | - | - | - | - | 1.17 | 7.0 | 6.0 |
| 29 | - | ○ | ○ | ○ | 1.15 | 10.8 | 3.1 |
| 30 | - | - | - | ○ | 1.04 | 11.0 | 1.9 |
| 31 | ○ | - | - | - | 1.11 | 11.1 | 5.4 |
| 32 | - | - | - | ○ | 1.05 | 9.6 | 4.3 |
| 33 | - | - | - | ○ | 1.00 | 8.2 | 3.4 |
| 34 | - | - | - | - | 1.12 | 8.1 | 3.5 |
| 35 | - | - | - | - | 0.89 | 10.6 | 1.3 |
| 36 | - | - | - | - | 1.10 | 7.2 | 6.3 |
| 37 | - | - | - | - | 1.17 | 10.4 | 1.8 |
| 38 | - | - | - | ○ | 1.09 | 7.9 | 6.5 |
| 39 | - | - | - | ○ | 0.85 | 8.1 | 1.2 |
| 40 | - | - | - | - | 1.08 | 7.4 | 5.6 |
| 41 | - | - | - | - | 1.02 | 7.7 | 7.3 |

ABI: ankle-brachial index; CAVI: cardio-ankle vascular index; FMD: flow-mediated dilation

**Table S4: Laboratory test results**

| n = 38 |  |  |  |  |  |  |  |  |  |
| --- | --- | --- | --- | --- | --- | --- | --- | --- | --- |
|  |  |  |  |  |  |  |  |  |  |
| Patient No. | TG (mg/dL) | HDL-C  (mg/dL) | LDL-C  (mg/dL) | HbA1c  ( NGSP, %) | FPG (mg/dL) | IRI  (μU/mL) | HOMA-R | HOMA-β  (%) | hsCRP  (mg/dL) |
| 1 | 56 | 80 | 113 | 5.4 | 94 | 1.9 | 0.4 | 21.8 | 2.74 |
| 2 | 46 | 82 | 81 | 5.4 | 80 | 0.7 | 0.1 | 14.2 | 0.05 |
| 3 | 161 | 40 | 103 | 9.5 | 126 | 5.1 | 1.6 | 29.1 | 2.50 |
| 4 | 179 | 60 | 123 | 6.3 | 152 | 12.5 | 4.7 | 50.6 | 0.32 |
| 5 | 117 | 71 | 129 | 7 | 103 | 11.6 | 3.0 | 104.4 | 10.20 |
| 6 | 119 | 41 | 123 | 6.5 | 146 | 7.8 | 2.8 | 34.0 | 0.15 |
| 7 | 49 | 49 | 134 | 6.1 | 132 | 3.9 | 1.3 | 20.2 | 0.07 |
| 8 | 90 | 64 | 67 | 6.6 | 102 | 4.1 | 1.0 | 37.6 | 3.94 |
| 9 | 53 | 65 | 64 | 6.1 | 96 | 1.3 | 0.3 | 13.9 | 0.08 |
| 10 | 183 | 49 | 75 | 7 | 228 | 44.4 | 25.0 | 96.9 | 19.90 |
| 11 | 68 | 60 | 88 | 7.2 | 168 | 18.9 | 7.8 | 64.8 | 1.12 |
| 12 | 201 | 64 | 132 | 6.2 | 128 | 11.6 | 3.7 | 64.2 | 0.50 |
| 13 | 154 | 48 | 183 | 6.8 | 121 | 2.8 | 0.8 | 17.1 | 0.40 |
| 14 | 65 | 68 | 80 | 6.8 | 135 | 2.3 | 0.8 | 11.5 | 0.07 |
| 15 | 118 | 51 | 148 | 6.9 | 140 | 23.1 | 8.0 | 108.0 | 0.50 |
| 16 | - | - | - | - | - | - | - | - | - |
| 17 | 185 | 43 | 111 | 7.7 | 140 | 6.9 | 2.4 | 32.2 | 0.86 |
| 18 | 120 | 52 | 129 | 5.8 | 94 | 5.5 | 1.3 | 64.2 | 0.65 |
| 19 | - | - | - | - | - | - | - | - | - |
| 20 | 63 | 87 | 104 | 5.6 | 99 | 1.8 | 0.4 | 18.4 | 0.15 |
| 21 | - | - | - | - | - | - | - | - | - |
| 22 | 152 | 47 | 165 | 8.3 | 165 | 8.4 | 3.4 | 29.7 | 3.64 |
| 23 | 157 | 39 | 140 | 6.7 | 119 | 6.2 | 1.8 | 39.7 | 1.82 |
| 24 | 187 | 52 | 115 | 7 | 136 | 5.9 | 2.0 | 29.2 | 0.37 |
| 25 | 66 | 60 | 114 | 5.4 | 102 | 5.1 | 1.3 | 46.8 | 0.12 |
| 26 | 117 | 40 | 141 | 6.3 | 132 | 6.0 | 2.0 | 31.3 | 0.32 |
| 27 | 305 | 75 | 154 | 6.3 | 111 | 3.0 | 0.8 | 22.7 | 0.12 |
| 28 | 43 | 120 | 69 | 4.9 | 92 | 4.7 | 1.1 | 58.8 | 0.66 |
| 29 | 114 | 62 | 142 | 6.3 | 128 | 4.5 | 1.4 | 25.1 | 0.21 |
| 30 | 76 | 73 | 155 | 6.8 | 139 | 6.5 | 2.2 | 30.8 | 0.61 |
| 31 | 115 | 64 | 134 | 7.1 | 160 | 3.4 | 1.4 | 12.7 | 0.09 |
| 32 | 190 | 46 | 111 | 5.7 | 91 | 9.3 | 2.1 | 119.8 | 7.67 |
| 33 | 58 | 63 | 112 | 6.4 | 115 | 5.8 | 1.7 | 40.3 | 0.30 |
| 34 | 96 | 54 | 182 | 5.8 | 100 | 2.7 | 0.7 | 25.8 | 0.15 |
| 35 | 45 | 71 | 118 | 5.3 | 88 | 0.7 | 0.2 | 10.2 | 0.16 |
| 36 | 276 | 53 | 229 | 5.7 | 99 | 5.6 | 1.4 | 55.6 | 1.24 |
| 37 | 65 | 38 | 54 | 6.2 | 94 | 0.8 | 0.2 | 9.1 | 13.30 |
| 38 | 75 | 72 | 167 | 6.3 | 119 | 4.1 | 1.2 | 26.2 | 0.33 |
| 39 | 122 | 56 | 85 | 7 | 110 | 1.1 | 0.3 | 8.3 | 4.69 |
| 40 | 97 | 101 | 100 | 5.5 | 96 | 1.7 | 0.4 | 18.4 | 0.52 |
| 41 | 144 | 53 | 113 | 5.5 | 88 | 1.6 | 0.4 | 23.6 | 2.32 |

TG: triglyceride; HDL-C: high-density lipoprotein cholesterol; LDL-C: low-density lipoprotein cholesterol; HbA1c: hemoglobin A1c;

FPG: fasting plasma glucose; IRI: immunoreactive insulin; HOMA-R: homeostasis model assessment ration; HOMA-β: homeostasis

model assessment of beta cell function; hsCRP: high-sensitivity C-reactive protein.
